# From Good to Bad: The Opposing Effects of PTHrP on Tumor Growth, Dormancy, and Metastasis Throughout Cancer Progression

**DOI:** 10.3389/fonc.2021.644303

**Published:** 2021-03-22

**Authors:** Courtney M. Edwards, Rachelle W. Johnson

**Affiliations:** ^1^Program in Cancer Biology, Vanderbilt University, Nashville, TN, United States; ^2^Vanderbilt Center for Bone Biology, Division of Clinical Pharmacology, Department of Medicine, Vanderbilt University Medical Center, Nashville, TN, United States; ^3^Division of Clinical Pharmacology, Department of Medicine, Vanderbilt University Medical Center, Nashville, TN, United States

**Keywords:** PTHrP, bone metastasis, dormancy, hypoxia, proliferation

## Abstract

Parathyroid hormone related protein (PTHrP) is a multifaceted protein with several biologically active domains that regulate its many roles in normal physiology and human disease. PTHrP causes humoral hypercalcemia of malignancy (HHM) through its endocrine actions and tumor-induced bone destruction through its paracrine actions. PTHrP has more recently been investigated as a regulator of tumor dormancy owing to its roles in regulating tumor cell proliferation, apoptosis, and survival through autocrine/paracrine and intracrine signaling. Tumor expression of PTHrP in late stages of cancer progression has been shown to promote distant metastasis formation, especially in bone by promoting tumor-induced osteolysis and exit from dormancy. In contrast, PTHrP may protect against further tumor progression and improve patient survival in early disease stages. This review highlights current knowledge from preclinical and clinical studies examining the role of PTHrP in promoting tumor progression as well as skeletal and soft tissue metastasis, especially with regards to the protein as a regulator of tumor dormancy. The discussion will also provide perspectives on PTHrP as a prognostic factor and therapeutic target to inhibit tumor progression, prevent tumor recurrence, and improve patient survival.

## Introduction

The initial discovery of parathyroid hormone- related protein (PTHrP) came about from studies on humoral hypercalcemia of malignancy (HHM), a paraneoplastic syndrome in which elevated levels of PTHrP lead to increased osteoclastic bone resorption and serum calcium levels (1, 2). HHM is most commonly diagnosed in patients with advanced-stage lung, renal, and neuroendocrine tumors. Though less frequently, breast cancers can also cause HHM. While hypercalcemia had been associated with cancer since the early twentieth century (3), it was Fuller Albright in 1941 who first postulated that this complication may be caused by tumor secretion of parathyroid hormone (PTH) or another similar factor due to its known roles in calcium homeostasis (4). While functionally this hypothesis seemed likely, clinically this did not prove to be true based on differences in the clinical presentation of patients with hypercalcemia due to cancer vs. those with hypercalcemia due to known PTH excess syndromes like primary hyperparathyroidism (5). By the 1980s multiple independent groups had eventually purified a protein similar in structure and biological function to PTH from human lung cancer (6), breast cancer (7), and renal cell carcinoma cell lines (8). This 18 kDA protein, now termed PTHrP, was found to have very high homology with the amino-terminal region of PTH such that eight of the first 13 residues are identical (9).

The role of PTHrP in cancer now extends well beyond its role in HHM. PTHrP is a well-established critical mediator of tumor-induced osteolysis, especially in breast cancer, which has a high tropism for disseminating to the bone marrow. In fact, ~70% of patients who succumb to breast cancer display evidence of bone metastases on postmortem examination (10). Lung cancer, melanoma, renal cell carcinoma, and thyroid cancers also metastasize to bone with relatively high (>20%) frequency (11) and form osteolytic lesions (12). Bone-disseminated tumor cells secrete PTHrP (13–15), which drives bone destruction *via* stimulation of receptor activator of NFκB ligand (RANKL)-mediated differentiation and activation of osteoclasts (16). Osteoclasts resorb the bone matrix, releasing numerous pro-tumorigenic factors such as transforming growth factor beta (TGFβ), matrix metalloproteinases, and other growth factors that subsequently fuel tumor cell proliferation and more PTHrP secretion (17, 18). PTHrP expression by bone-disseminated tumor cells is also uniquely fueled by the microenvironment. The rigidity of the bone matrix activates TGF-β dependent mechanical signals that stimulate expression of both PTHrP and Gli2, a transcription factor in the hedgehog signaling pathway that in turn induces more PTHrP expression (19). The bone microenvironment provides yet another critical level of regulation of Gli2 and PTHrP expression *via* the Wnt pathway (20). Matrix rigidity activates Wnt signaling and induces nuclear β-catenin accumulation, while bone marrow stromal cells secrete canonical (including Wnt3a) and non-canonical Wnt ligands. Both processes further drive Gli2 and PTHrP transcription and eventual bone destruction.

Patients who develop bone metastases may experience severe pain, impaired mobility, pathologic fractures, spinal cord compression and hypercalcemia (21). While bisphosphonates and denosumab, inhibitors of osteoclast activity, are commonly utilized to manage metastasis-related symptoms and have been shown to decrease the incidence of bone metastases, they only improve survival in postmenopausal breast cancer patients (22, 23). No survival benefits have been observed in patients who were premenopausal at the time of diagnosis (24–26). Thus, there remains an urgent need to identify therapies that effectively target bone metastatic tumors and improve survival.

In addition to its critical role in tumor-induced bone disease, PTHrP has more recently been investigated as a potential regulator of tumor dormancy owing to its roles in regulating tumor cell proliferation, apoptosis, and survival (27) and genes that have been specifically implicated in tumor dormancy (28). Generally, dormancy can be characterized as (i) cellular dormancy where tumor cells persist as either solitary cells that are non-proliferative (e.g., Ki67 or BrdU negative) and arrested in the G0 cell cycle phase, or (ii) tumor mass dormancy where the growth capacity of micrometastases is limited due to balanced proliferation and apoptosis, suppression of angiogenesis, or immune clearance (29–31). Tumor dormancy is believed to be the cause of late recurrence even months to years following successful removal of the primary tumor (32–34). Long latency periods are mostly frequently observed in estrogen-receptor positive (ER^+^) breast cancer (35, 36) as well as prostate cancer (37). Patients with other cancer types including non-small cell lung cancer (38), renal cell carcinoma (39) and colorectal cancer (40) may also exhibit late tumor recurrence, although this occurs less frequently than in breast cancer patients. Dramatic increases in breast cancer patient survival in recent decades are, in part, due to significant improvements in treating and managing primary breast tumors. Thus, it is possible that as therapies continue to improve for other cancer types that extend patient survival, longer latency periods may also be observed. Tumor dormancy presents a significant clinical dilemma as these dormant DTCs not only have the potential to become reactivated and proliferate into a macrometastasis, but also evade immune surveillance and anticancer therapy (41, 42). Currently there are no cures for metastatic disease or therapies to prevent tumor exit from dormancy and late recurrence. This review will explore current knowledge from preclinical and clinical studies regarding the role of PTHrP in promoting skeletal and soft tissue metastasis. We will also explore findings regarding PTHrP as a regulator of tumor dormancy and provide our perspective on PTHrP as a therapeutic target to inhibit tumorigenesis, prevent tumor recurrence, and improve patient survival.

## PTHrP Biology

*PTHLH*, the gene encoding PTHrP, is located on chromosome 12, and has nine exons spanning ~15 kb with at least three identified promoters. Alternative splicing gives rise to three isoforms containing 139, 141, and 173 amino acids (43). PTHrP also has multiple domains, each with different biological functions (44). The first 36 amino acids (−36 to −1) encode a domain that controls intracellular trafficking of PTHrP precursors before being cleaved when the mature molecule is secreted. The next domain (amino acids 1–34) is responsible for PTHrP binding to and activation of the PTH receptor type 1 (PTH1R), a G-protein coupled receptor. In fact, eight of the first 13 residues within this region of PTHrP are identical with PTH, allowing the two polypeptides to exert agonist effects on their shared receptor (44, 45). The nuclear localization sequence (NLS) from amino acids 67–94 is important for intracrine actions of PTHrP in the nucleus and cytoplasm including regulating cell proliferation, survival, and apoptosis (46). Lastly, the carboxy-terminal domain beginning at residue 107 is associated with a number of identified biological actions including inhibition of osteoclast-mediated bone resorption and anabolic effects in bone *via* a region termed “osteostatin” as well as a nuclear export sequence (NES) (43, 47).

### Endocrine, Autocrine, and Paracrine Activity of PTHrP

In normal physiology, PTHrP acts as a hormone to control calcium transport across the placenta to the fetus (48) and during lactation when it enters systemic circulation (49). In HHM, PTHrP secreted by tumors in the breast and lung, for instance, also acts as a hormone distantly to increase bone resorption (1, 2). The autocrine and paracrine roles of PTHrP in normal postnatal physiology have been reviewed extensively elsewhere (44, 46). Thus, several of these physiologic functions that are less pertinent to this review will only briefly be mentioned here. PTHrP is highly expressed in human tissues and plays important roles in mammary gland development, tooth eruption, keratinocyte differentiation for hair follicle development, chondrocyte maturation, and endochondral bone formation (44, 46). Perhaps one of the most well-studied paracrine functions of PTHrP is the regulation of normal bone remodeling where it is produced locally by early osteoblast progenitors to promote differentiation of mature osteoblasts and bone formation (50, 51). PTHrP also inhibits apoptosis of early and mature osteoblasts and osteocytes. Furthermore, osteoblast-derived PTHrP stimulates osteoclast differentiation to increase bone resorption. These actions of PTHrP must occur in a balanced manner to maintain the integrity of the bone. While physiologic, these paracrine functions of PTHrP can also pathologically fuel osteolysis and the growth of bone disseminated tumors as discussed previously (17, 18). Lastly, PTHrP plays a well-recognized role as a paracrine regulator of smooth muscle relaxation, particularly in the vasculature (52) where incubation with PTHrP (1–34aa) also activates cAMP production, indicating that this effect is indeed mediated through PTH1R (53, 54). In vascular smooth muscle cells, treatment with exogenous PTHrP acting through PTH1R inhibits cell proliferation (55, 56).

In addition to binding and activating PTH1R to exert its paracrine/autocrine functions, PTHrP can translocate into the nucleus when its NLS forms a complex with importin β, a nuclear transport factor, and the GTP-binding protein Ran (57). Interestingly, in vascular smooth muscle cells, intracrine actions of PTHrP localized to the nucleus paradoxically increase proliferation (56). Indeed, in A10 smooth muscle cells overexpressing wild-type PTHP, the protein localizes in the nucleus of cells that are dividing or completing cell division. This is in striking contrast to findings that PTHrP inhibits proliferation and cell cycle progression in the same cells when acting through PTH1R (55, 56). These effects of PTHrP are particularly important in the discussion of its role as a regulator of tumor dormancy as it has also been demonstrated that PTHrP lacking the NLS arrests cell cycle progression by increasing p27^Kip^, a cyclin dependent kinase inhibitor, and decreasing phosphorylation of Rb (58, 59). Cell cycle arrest in the G0-G1 phase is a key characteristic of quiescent cells (60, 61) and p27 is elevated in G0 arrested cells (62, 63). These findings in vascular smooth muscle cells are remarkable as they indicate that PTHrP can have paradoxical roles on mitogenesis depending on the mode of signaling: paracrine/autocrine vs. intracrine.

In addition to nuclear localization mediated by importin β, PTHrP can also gain entry into the nucleus by other mechanisms. PTHrP can be secreted but then internalized in an autocrine/paracrine manner *via* the PTH1R before being shuttled to the nucleus (64). Secreted PTHrP may also enter the nucleus through endocytosis-dependent translocation initiated by binding with a non-PTH1R cell surface receptor (65). Another potential mechanism regulating its subcellular localization is if translation is initiated at a codon different from the classic AUG site. As a known example, translation of fibroblast growth factor-3 (FGF3) can be initiated at an AUG codon resulting in direction of the peptide for secretion (66). If translation begins at an alternative upstream CUG site, FGF3 is directed into the nucleus. Like FGF3, the PTHrP prepro region has an alternative translational start site at a CUG codon (67), which may serve a similar purpose in regulating PTHrP secretion vs. nuclear import. Since the differential localization of PTHrP produces divergent mitogenic cellular effects in vascular smooth muscle cells, the same is likely true in cancer cells, complicating the understanding of PTHrP as a regulator of cell proliferation and tumor dormancy. Consequently, if altering PTHrP nuclear localization is to be leveraged for therapeutic purposes, more investigation is needed to better understand the regulation of PTHrP subcellular localization in cancer cells and how this may change throughout tumorigenesis.

## Roles of PTHrP in Tumorigenesis, Metastasis, and Tumor Dormancy

### Preclinical Evidence for PTHrP Regulation of Tumor Growth and Proliferation

Our understanding of the paracrine/autocrine and intracrine actions of PTHrP extends far beyond the physiologic activities described in the bone, vasculature, and various other normal epithelial tissues. PTHrP also modulates growth, progression, and metastasis in various cancer types by regulating: (i) cell survival, (ii) cell proliferation, (iii) apoptosis, and (iv) invasion and migration (68, 69). For example, human MCF7 breast cancer cells overexpressing PTHrP (−36 to −139) display significantly greater survival as they are protected from serum starvation-induced apoptosis and express elevated levels of the antiapoptotic proteins Bcl-2 and Bcl-xL (69). Other studies have demonstrated that PTHrP drives breast tumor growth by promoting proliferation, as demonstrated by increased staining for the proliferative markers Ki67 and cyclin D1 (61). Human breast cancer cells expressing PTHrP (−36 to −139) are also enriched in the G2/M cell cycle phase compared with cells overexpressing NLS-mutated PTHrP, indicating an intracrine role for PTHrP in regulating cell cycle progression and cell growth. In prostate cancer cells, PTHrP expression stimulates proliferation and induces intracrine production of Il-8, a known growth-promoting factor (70). Prostate cancer cells overexpressing full-length PTHrP also show significantly increased cell survival when exposed to various apoptotic agents (71). Another study determined that treatment with PTHrP neutralizing antibodies dramatically inhibits clear cell renal cell carcinoma cell proliferation *in vitro* and induces regression of implanted tumors by inducing apoptosis *in vivo*, further indicating a role for PTHrP in regulating both proliferation and cell death (72). PTHrP also positively regulates LoVo human colon cancer cell proliferation, migration and invasion *in vitro* (73). Lastly, human cancer cells that overexpress full-length PTHrP display upregulated expression of the α1, α5, α6, and β4 integrin subunits (74), which are known to facilitate cancer cell adhesion, migration and invasion (75, 76).

PTHrP expression in the primary tumor has also been identified as an important regulator of tumor growth in *in vivo* genetic models. In the PyMT-MMTV (mouse mammary tumor virus-polyoma middle T antigen) model of breast carcinoma where mice spontaneously develop mammary tumors, Cre-loxP-mediated *Pthlh* ablation delays primary tumor initiation and inhibits tumor progression (68). Palpable tumors appear much later and measure smaller than those in control mice. Mechanistically, the authors found reductions in the expression of Ki67, factor VIII (an angiogenesis marker), Bcl-2 (an antiapoptotic protein), cyclin D1 (a cell-cycle regulator), and AKT1 (a pro-survival factor). These data indicate that in this model of breast cancer, PTHrP acts as a pro-tumorigenic factor that drives tumor cell growth and proliferation in the primary site. In striking contrast, another *in vivo* study found that Cre-mediated loss of PTHrP in the MMTV-*neu* mouse model increases tumor incidence and reduces survival (77). In comparing these discrepant results from the studies on the PyMT-MMTV mice (68) vs. the MMTV-*neu* mice (77), it is important to note that the *neu*-based model reflects late-onset oncogenesis representing tumors arising in older animals while the PyMT-MMTV-based model reflects earlier onset tumorigenesis. Age can significantly affect tumor behavior (78, 79). Thus, in these pre-clinical tumor models, age at which cancer develops must be carefully factored into the interpretation of the effects of PTHrP on tumorigenesis. Lastly, authors of the PyMT-MMTV study report that they deleted exon 4, which encodes amino acids 1–137 in mice (44). While the authors of the MMTV-*neu* study do not explicitly state which portion of the gene was targeted, deletion of a different exon or smaller portion of the gene could explain these opposing observations since targeting different domains of the PTHrP molecule can elicit distinct cellular responses.

The previously discussed *in vivo* studies all rely on mouse tumor models. However, it is important to also recognize the utility of studies investigating PTHrP using models of spontaneous cancers that develop in larger animals such as dogs and cats (80). These animal models also contribute to our understanding of the biology of PTHrP and its role in tumorigenesis in ways that are distinct from studying rodent models alone. For instance, in feline oral squamous cell carcinoma (OSCC), elevated expression of PTHrP [which displays a high degree of sequence similarity to human isoforms (81)] is associated with increased bone invasion and osteoclastogenesis (82). Interestingly, tumor cells derived from bone specimens with evidence of osteolysis have more nuclear localization of PTHrP compared to OSCC without osteolysis. This model provides a unique finding where in addition to the paracrine actions of PTHrP in the bone, the intracrine functions of the polypeptide also appear to strongly influence the osteolytic phenotype of tumor cells. A feline oropharyngeal squamous cell carcinoma cell lines (SCCF1) has also been developed that expresses elevated PTHrP mRNA and protein in response to TGF-β stimulation (83) similar to human cancer cell lines (19, 84). Elevated PTHrP expression has also been noted in numerous neoplastic canine tissues compared with normal matched tissue (85, 86). There is great potential to learn even more about the biology of PTHrP and its role in tumorigenesis using these large animal models.

### PTHrP's Role in Regulating Tumor Cell Dormancy

Most pre-clinical data support a pro-tumorigenic role for PTHrP. PTHrP is also likely a negative regulator of tumor cell dormancy due to its actions that modulate proliferation, apoptosis and cell survival. One study that provides some of the most direct and striking evidence to support this found that in ER^+^ human MCF7 breast cancer cells, which lie dormant *in vivo* following intracardiac injection (28, 87–89), overexpression of PTHrP (1–141) pushes these cells out of quiescence, switches them to a highly osteolytic phenotype and dramatically increases tumor burden in the bone (87). Consistent with this enhanced bone colonization and exit from dormancy, a later study determined that PTHrP (1–139) overexpression in MCF7 cells also represses expression and downstream signaling of leukemia inhibitory factor receptor (*LIFR*), a known breast tumor suppressor and dormancy factor in the bone (28). In this study, overexpression of PTHrP and loss of LIFR both enable otherwise dormant breast cancer cells to downregulate several quiescence- associated genes including thrombospondin-1 (TSP1) (90), transforming growth factor-β2 (TGF-β2) (91), tropomyosin-1 (TPM1) (92), and Selenbp1 (93), among others. Common regulation of this group of genes suggests that PTHrP may inhibit pro-dormancy signaling mediated by LIFR. Moreover, intracardiac injection of MCF7 LIFR knockdown cells into mice results in greater bone destruction *via* increased osteoclastogenesis and tumor cell proliferation (28). Thus, repression of LIFR either directly or perhaps through PTHrP overexpression can push bone-disseminated breast tumor cells out of dormancy. These data are further supported by the PyMT-MMTV genetic studies by Li et al. (68), which demonstrated that *Pthlh* ablation reduces primary breast tumor growth with reductions in pro-proliferative factors Ki67 and cyclin D1 as well as the anti-apoptotic protein Bcl-2, all factors known to regulate dormancy.

Interestingly, evidence exists suggesting that multiple breast cancer cell lines express PTH1R at varying levels, but do not activate downstream cAMP signaling in response to PTH or PTHrP, despite functional signaling in response to calcitonin and PGE_2_ which serve as positive controls (94). In this study, there was also no activation of a cAMP response element reporter construct, and RNA sequencing confirmed that only 2 out of 36 genes in a previously described panel of cAMP responsive element binding protein (CREB)-responsive genes (95) were significantly upregulated in MCF7 PTHrP-overexpressing cells. Taken together, these data provide convincing evidence that in the bone colonization models, the effects of PTHrP overexpression on gene expression, including dormancy-associated factors in MCF7 cells, are independent of PTH1R activation of the cAMP/PKA/CREB pathway. Further studies are warranted to explore non-PTH1R mediated actions, which may reveal novel mechanisms by which PTHrP negatively regulates dormancy in bone-disseminated breast tumor cells.

Lastly, other studies in breast cancer have also revealed that PTHrP may alter adhesion to extracellular matrix (ECM) cell surface receptors, which can trigger intracellular signaling that promotes cell cycle progression and exit from a dormant state (96–98). Specifically, PTHrP regulates the expression of integrins which mediate interactions between tumor cells and the ECM that can modulate cellular quiescence (99). For example, downregulation of the urokinase plasminogen activator receptor (uPAR), a known mediator of tumor dormancy *in vivo*, decreases complex formation with α5β1 integrin and cell adhesion to fibronectin (100). This reduced ECM binding consequently maintains tumor cells in a dormant state by inhibiting activation of extracellular regulated kinase (ERK) signaling, which normally functions to promote cell cycle progression and division (101). Additional studies have also confirmed that inhibiting ERK signaling *via* altered uPAR-mediated α5β1 integrin interactions promotes quiescence *in vivo* (102). This is highly relevant in the evaluation of PTHrP as a regulator of dormancy since overexpression of PTHrP (−36 to −139) in MDA-MB-231 human breast cancer increases adhesion to fibronectin (103). PTHrP (−36 to −139) overexpression in tumor cells also significantly increases mRNA and cell surface expression of various integrins including α_5_, α_6_, β_1_, and β_4._ Though it has not been directly studied, PTHrP may push tumor cells out of dormancy by inducing integrin expression, cell adhesion to fibronectin, and activation of ERK signaling. Additional studies are needed to understand how PTHrP alters ECM binding to regulate tumor dormancy.

Prostate tumors, like breast tumors, also exhibit long latency periods before micrometastases become clinically detectable (37, 92). One study found direct evidence that PTHrP promotes prostate cancer progression in the bone (104). Overexpression of PTHrP (1–87) and PTHrP (1–173) in the non-invasive DU-145 human prostate cancer cell line converted these cells to an aggressive phenotype resulting in significantly greater bone tumor burden and mixed osteolytic/osteoblastic lesions following intrafemoral injection. Interestingly, mice injected with PTHrP (1–173) cells had more extensive bone lesions than those injected with PTHrP (1–87) mice despite lower serum PTHrP levels. Not only does this study demonstrate that PTHrP expression can push prostate tumor cells out of dormancy but it also highlights the pleiotropic actions of the protein's different domains, as PTHrP (1–87) lacks the full nuclear localization sequence, osteostatin region, and mitogen regulatory sequences contained in the carboxy terminus of the full-length molecule. The effects of the carboxy terminus of PTHrP, in particular, need to be examined more extensively to specifically understand how this region promotes cancer progression in bone and regulates tumor dormancy. Another study of early prostate adenocarcinoma also demonstrated that PTHrP overexpression significantly increases primary tumor growth (105). This study found no difference in growth rates between human prostate cancer cells transfected with full-length PTHrP and vector controls, but PTHrP overexpression did render the cells less susceptible to phorbol-12-myristate-13-acetate (PMA)- induced apoptosis. Other studies have also identified a role for PTHrP in inhibiting apoptosis (106, 107). Thus, PTHrP may negatively regulate tumor dormancy by not only increasing cell proliferation, but also by disrupting the balance with cell death.

Interestingly, other *in vitro* studies, particularly on tumor cells in soft tissues have provided contrasting findings on the role that PTHrP plays in tumor dormancy. Administration of neutralizing antibodies against PTHrP (1–34) to mice inoculated with PTHrP-expressing orthotopic lung carcinomas significantly increases tumor growth (108). In a later study by the same authors on human lung adenocarcinoma lines that are normally PTHrP-negative, ectopic expression of PTHrP (1–87) induces arrest in or slows progression through G1 compared with control cells (109). Expression of cyclin D2 and cyclin A2 were also lower while expression of p27^Kip1^, a cyclin-dependent kinase inhibitor, was increased indicating that PTHrP inhibits the proliferation of lung tumor cells and may actually promote dormancy in this tumor model. It is interesting to note that in this study, as in the breast cancer study by Johnson et al. (94) discussed previously, there was no observed increase in cAMP production, making autocrine/paracrine signaling *via* PTH1R unlikely. In addition, the plasmid for PTHrP (1–87) encodes a truncated protein lacking the full NLS suggesting that extra-nuclear forms of the protein may interact with other cytoplasmic factors to regulate tumor cell proliferation. However, it is worth noting that peptides <50–60 kDA such as PTHrP (1–87) can still passively enter the nucleus without an NLS (110). Thus even truncated forms of PTHrP that lack the NLS may still localize to the nucleus. This further highlights the necessity of more studies to establish whether the mitogenic and dormancy effects of PTHrP depend on autocrine/paracrine, or intracrine mechanisms.

The studies on breast, prostate and lung cancer discussed in the previous sections do present mixed findings regarding the role of PTHrP in regulating tumor growth and dormancy. This would suggest that the actions of PTHrP are highly dependent on the tumor type and microenvironment. In the bone, tumor cell autonomous actions of PTHrP promote emergence from a quiescent state (28, 87, 104). This may be complemented by paracrine actions of tumor-secreted PTHrP on bone marrow stromal cells like osteoclasts that promote the release of additional pro-tumorigenic factors to further increase tumor growth. However, in tumors and metastases that arise in other soft tissues, the opposite may be true. This is also evident in another *in vivo* small cell lung cancer study where administration of an anti-PTHrP antibody significantly inhibits bone metastasis formation, but not metastasis to visceral organs (lungs, liver, kidneys, lymph nodes) (14). This suggests that PTHrP may uniquely drive metastasis formation in the bone, but not other soft tissues. Clinical evidence of PTHrP's unique role in metastasis to bone vs. soft tissues will be discussed further in later sections. This is particularly important as the potential success of PTHrP targeted therapies will depend on careful selection of patients with tumor types at highest risk for recurrence in organs where its expression actually drives exit from dormancy and metastatic outgrowth.

### PTHrP's Role in Regulating Tumor Mass Dormancy

In addition to modulating cellular dormancy, PTHrP's role in regulating angiogenesis and immunosurveillance, the two key mechanisms that characterize tumor mass dormancy, must also be considered. Angiogenesis is critical as tumors generally cannot exceed 2–3 mm in diameter without developing new blood vessels or co-opting pre-existing vasculature to avoid growth-limiting oxygen deprivation due to hypoxia (low oxygen tensions) and nutrient deprivation (111). Importantly, the bone marrow is a physiologically hypoxic microenvironment (112, 113) and hypoxia is evident in most solid tumors (114). Angiogenic dormancy results when insufficient vascularization induces cell death that counterbalances the rate of proliferation, resulting in no net growth of the tumor mass (60, 115). Emergence from dormancy and tumor progression may resume after an “angiogenic switch” in which there is a shift in the balance between pro-angiogenic factors [e.g., vascular endothelial growth factor (VEGF)] and anti-angiogenic factors (e.g., thrombospondin-1) (116). Consequently, pro-angiogenic signaling dominates and new blood vessels form.

Several studies have investigated the effects of PTHrP on tumor-induced angiogenesis, though the results are conflicting. Early work by Bakre et al. demonstrated that PTHrP inhibits endothelial cell migration *in vitro* and angiogenesis in prostate tumors *in vivo* through activation of protein kinase A (117). Consistent with this inhibitory effect, PTHrP reduces VEGF production during osteoblast differentiation and endochondral bone formation (118). These results suggest that PTHrP may prevent tumor growth by inducing angiogenic dormancy. However, numerous other studies have demonstrated that PTHrP stimulates tumor-induced angiogenesis. PTHrP increases expression of pro-angiogenic factors including VEGF (119), and factor VIII (68) in breast cancer bone metastases. In prostate cancer cells PTHrP overexpression stimulates IL-8 production, another key pro-angiogenic factor (70). Malignant pituitary tumor cells that overexpress PTHrP also induce neovascularization in xenografts *in vivo* (120). Mechanistically, recombinant PTHrP (1–34) increases capillary formation by endothelial cells through PTH1R activation and cAMP signaling. Another study found that exogenous PTHrP treatment *in vivo* increases expression of VEGF and CD31 (a marker of vascular endothelial cells) in colorectal tumors (118).

Overall, these studies indicate that PTHrP promotes tumor-induced angiogenesis, making it plausible that the protein could act as a key negative regulator of tumor dormancy by stimulating new vessel formation. Conflicting findings are likely due to diversity within the tumor microenvironment where there are different target cells of PTHrP that each may individually regulate angiogenesis. Moreover, different domains and biologically active fragments of PTHrP likely will have differing effects on endothelial cells and other stromal cells during angiogenesis, but these studies did not explore differences between the different PTHrP isoforms. Lastly, it is important to note that while angiogenesis and angiogenic dormancy can be regulated by both hypoxia and PTHrP activity, PTHrP is also regulated by hypoxic signaling. Studies in chondrocytes determined that PTHrP expression is induced by hypoxia in a HIF1α (hypoxia inducible factor 1 alpha) and HIF2α (hypoxia inducible factor 2 alpha) dependent manner (121). However, it has been show in prostate cancer cells that while HIF1α and HIF2α are both able to bind to the *PTHLH* promoter, only HIF2α induces transcription (122). As hypoxia has been shown to have dual roles in both promoting and negatively regulating quiescence (123, 124), PTHrP's complex role in angiogenesis may be yet another mechanism by which low oxygen tensions differentially regulate tumor dormancy.

Immunosurveillance plays a well-characterized role in suppressing tumor growth and maintaining micrometastases in a dormant state (125). Components of the adaptive immune system including CD4^+^ (126, 127) and cytotoxic CD8^+^ (128) T cells are key players known to limit the outgrowth of dormant disseminated tumor cells (129). Natural killer (NK) cells are a pivotal component of the innate immune system that can maintain tumors in a dormant state by both their cytotoxic activity as well as stimulation of anti-tumorigenic cytokine production by CD4^+^ and CD8^+^ T cells (130, 131). In contrast, regulatory T cells (Tregs) are associated with immune suppression and tumor progression in numerous cancer types (132, 133). Lastly, the myeloid-derived suppressor cells (MDSCs) are a unique subpopulation of immature myeloid cells that play a prominent role in reactivating dormant disseminated tumor cells and promoting metastatic outgrowth by promoting immune suppression and angiogenesis (134, 135). While few studies have examined the role of PTHrP in modulating tumor infiltration of each of these immune cell types, a few have specifically examined the MDSCs that are identified by the expression of myeloid cell (CD11b) and granulocytic (Gr-1) markers (136). One study found that treatment with recombinant PTHrP or overexpression of the protein both promote the recruitment of CD11b^+^Gr1^+^ MDSCs into prostate tumor tissue where they increase primary tumor growth *in vivo* (137). In the bone marrow, tumor-derived PTHrP also promotes recruitment and activation of CD11b^+^Gr1^+^ MDSCs, resulting in increased MDSC-derived MMP-9 expression, which drives prostate cancer invasion and angiogenesis. Similar findings were demonstrated in a separate study of murine mammary carcinoma where intratumoral CD11b^+^Gr1^+^ cell recruitment enhanced metastatic outgrowth *via* increased metalloproteinase activity (138). CD11b^+^Gr1^+^ MDSCs derived from the bone marrow of breast tumor-bearing mice also have elevated expression of transforming growth factor β (TGFβ), a well-known potent stimulator of PTHrP expression, thus perpetuating the cycle of tumor-induced osteolysis that fuels tumor growth (139). Taken together, these results suggest that PTHrP may play a critical role in negatively regulating tumor mass dormancy by increasing infiltration of immune suppressive MDSCs, which promote tumorigenesis (140, 141). PTHrP actions on recruitment of other immune populations in the tumor microenvironment have been inadequately explored. These studies are critical to gaining a more complete understanding of the role of PTHrP as a regulator of tumor mass dormancy.

### Clinical Evidence for PTHrP Effects on Tumor Growth and Metastasis

Much like the *in vitro* and *in vivo* analyses, clinical studies investigating PTHrP as a prognostic factor have produced opposing findings, complicating the understanding of the role of the molecule in tumorigenesis, metastasis, and tumor dormancy. Henderson et al. conducted a large and comprehensive prospective study over 10 years in patients with breast cancer and found that positive immunohistochemical staining for PTHrP in 79% of the primary tumors was associated with significantly improved survival and decreased bone metastasis (142). These results would suggest that PTHrP decreases the invasive capacity of breast tumor cells and is protective against tumor growth in the primary site and formation of distant metastases. Interestingly, this study also revealed that of the 19 patients with bone metastases requiring surgical intervention, 7 patients had PTHrP-negative primary tumors. However, the majority of the individuals with PTHrP-negative primary tumors still developed PTHrP-positive bone lesions. All patients in the study with PTHrP-positive primary cancers also had positive expression in their bone metastases. Thus, there is not a clear inverse relationship between PTHrP expression at the primary and bone secondary sites. It is important to note this frequency of bone metastases in patients with PTHrP-negative primary breast cancers is still consistent with known tumorigenic roles for PTHrP when tumor cells colonize the bone later in disease progression. The bone marrow microenvironment enhances tumor cell production of PTHrP, which drives osteolysis and further metastatic growth (17, 18). Thus, protective PTHrP actions early in tumorigenesis at the primary site are likely distinct from its deleterious effects once disseminated tumor cells reach the bone.

Another breast cancer study that aligns with the overall conclusions of Henderson et al. (142) found that PTHrP levels are downregulated in malignant compared with normal breast epithelia, but also low levels of nuclear localized PTHrP correlate with unfavorable clinical outcomes (143). Mechanistically, the authors found a strong positive correlation between nuclear PTHrP levels and nuclear pStat5. This may explain, in part, why nuclear PTHrP is associated with the unfavorable clinical outcomes since loss of Stat5 expression and activation in breast cancer has consistently been associated with poor prognosis (144, 145). Again, this observed progressive loss of nuclear PTHrP from well-differentiated mammary epithelia to poorly differentiated, aggressive cancer cells would suggest important context-dependent roles for PTHrP signaling in tumorigenesis. In early stages, intracrine signaling of nuclear PTHrP may be protective against malignant transformation, but in distant sites like the bone, reactivation of PTHrP can still induce extensive osteolysis that would drive metastatic tumor growth.

Other clinical studies have identified protective roles of PTHrP in other solid tumor types. An analysis of non-small cell lung cancer (NSCLC) determined that PTHrP (109–141) expression in the primary tumor was associated with longer disease-free survival in female patients with either early or advanced stages of disease (146). Interestingly, female patients in this study with PTHrP-negative cancer had a shorter lifespan than all other participants, including male patients with PTHrP-negative or positive carcinomas. Thus, absence of tumor PTHrP appears to be a negative prognostic indicator specifically in women with NSCLC. The exact etiology of the sex dependence of PTHrP as a prognostic factor in lung cancer has not been further studied. However, 17β-estradiol (E_2_) and tamoxifen have both been shown to regulate PTHrP expression in breast cancer cells (147), suggesting an association between estrogen receptor signaling and PTHrP during tumorigenesis. Lastly, in clear cell renal cell carcinoma (RCC), it has also been determined that decreased intensity of the carboxy-terminal region of PTHrP (amino acids 109–141) is associated with significantly greater cases of tumor recurrence (148). This would suggest a role for PTHrP in increasing recurrence free survival in patients with RCC.

By contrast, numerous other clinical studies, especially in breast cancer, have concluded that PTHrP supports tumor growth and progression. In a large analysis including two genome-wide association studies from 41 case–control studies through the Breast Cancer Association Consortium (BCAC) and nine breast cancer genome-wide association studies, *PTHLH* was identified as a susceptibility locus in both ER^+^ and ER^−^ breast cancer (149). This study of patients with invasive breast cancer and ductal carcinoma *in situ* (DCIS) provides additional evidence implicating PTHrP in breast cancer pathogenesis, independent of its roles in promoting osteolysis. It is important to note that this analysis was performed on data from retrospective case-control studies enrolling multiple smaller patient cohorts. This factor should be kept in mind when comparing these findings with those of the better-powered, prospective study conducted by Henderson et al. (142) that identified PTHrP as a protective factor. In another study on patients with ER^+^ and ER^−^ breast cancer, expression of both PTHrP and its receptor correlated with reduced disease-free survival while receptor expression alone correlated with reduced overall survival (150). In this study, PTHrP expression was detected by an antibody to the amino terminal region (1–34) in 68% of primary tumor specimens compared with 100% of bone metastases and the PTHrP receptor was present in 37% of tumors compared with 81% of bone metastasis samples. Thus, PTHrP and its receptor are expressed more frequently in bone metastases than primary tumors. However, the functional relevance of this pattern of receptor expression in bone-disseminated tumor cells is still unclear since *in vitro* data indicate that in ER^+^ breast cancer cells, activation of PTH1R/cAMP signaling does not regulate dormancy gene expression (95). Nevertheless, while expression of the receptor may not regulate dormancy in the bone, these clinical data still support the understanding that PTHrP expression by bone disseminated tumor cells is critical to their ability to establish metastatic colonies and possibly promote exit from dormancy. Other studies have also confirmed a positive association between PTHrP expression in primary breast tumors and bone metastasis as well as shortened overall survival (151, 152).

Though in a different metastatic site, a recent study on early stage triple negative breast cancer (TNBC) found that PTHrP expression is significantly correlated with decreased central nervous system (CNS)-progression free survival (153). These findings, if validated in other large cohorts of early-stage, newly diagnosed TNBC patients, would raise the hypothesis that monitoring PTHrP expression in TNBC patients could detect the initial stages of CNS metastasis and identify individuals with recurrent tumors much earlier than conventional detection techniques. Interestingly, this study did not identify a statistically significant relationship between PTHrP expression and the incidence of bone metastasis. It is important to note that only specimens from patients with early stage TNBC without evidence of metastasis at presentation or multiple primary malignancies were analyzed. Thus, examination of patients with later staged cancer may also reveal a significant association between PTHrP expression and bone metastasis in TNBC. This highlights the importance of examining patients with all subtypes of breast cancer and stages of disease progression when investigating PTHrP as a prognostic factor for metastasis and late recurrence.

Clinical evidence also exists suggesting a role for PTHrP in tumor growth and metastasis in other tumor types. In prostate cancer, PTHrP expression varies depending on the cancer stage, with expression detected in 33% of benign prostate hyperplasias, 87% of well-differentiated tumors and 100% of poorly differentiated and metastatic tumors (154). Other studies have similarly found that PTHrP is expressed in prostatic bone metastases (155). Here it seems that a progressive gain of PTHrP in disease progression is associated with tumorigenesis and distant metastasis. In a study of patients with early-stage lung adenocarcinoma, positive staining for PTHrP (1–34) is associated with worse overall survival and metastasis-free survival, independent of tumor stage (156). Survival is even more dramatically reduced in patients with tumors co-expressing high levels of N-terminal PTHrP and PTH1R. Taken together, these observations would suggest that paracrine/autocrine mechanisms involving PTHrP may drive tumor progression in lung cancer.

## Authors Perspectives on PTHrP as a Prognostic Factor and Dormancy Regulator

**Figure 1:**
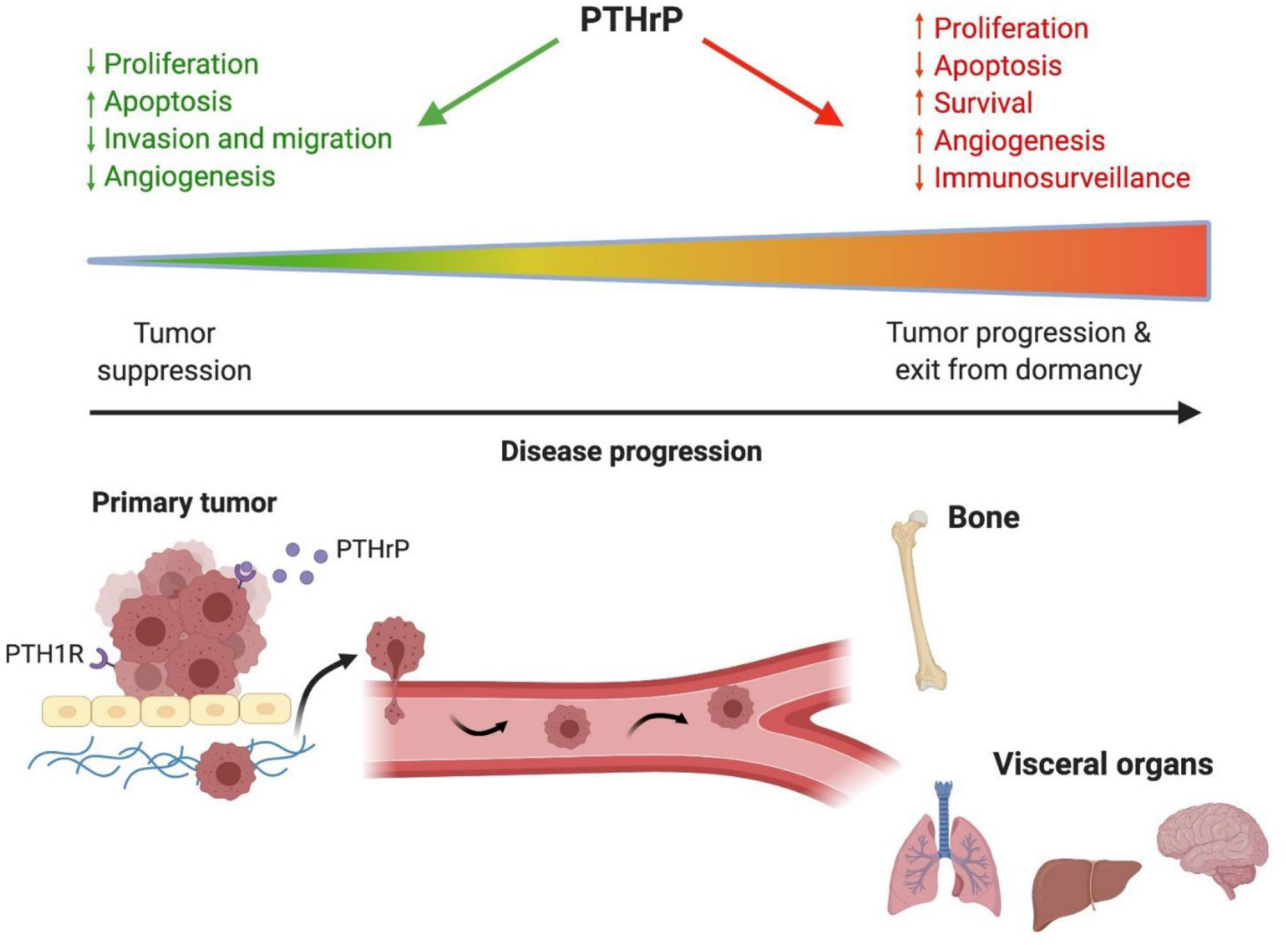
PTHrP has different actions throughout cancer progression. Early in tumorigenesis PTHrP is protective against tumor formation in the primary site by decreasing proliferation, promoting apoptosis, decreasing angiogenesis and reducing tumor cell invasion and migration. Late in disease progression when tumor cells disseminate to distant sites, PTHrP promotes tumor progression and exit from dormancy by stimulating proliferation and angiogenesis while reducing apoptosis and immunosurveillance. These actions in advanced stages of disease contribute to poor patient outcomes and reduced survival. PTHrP, parathyroid hormone-related protein.

**Figure 2:**
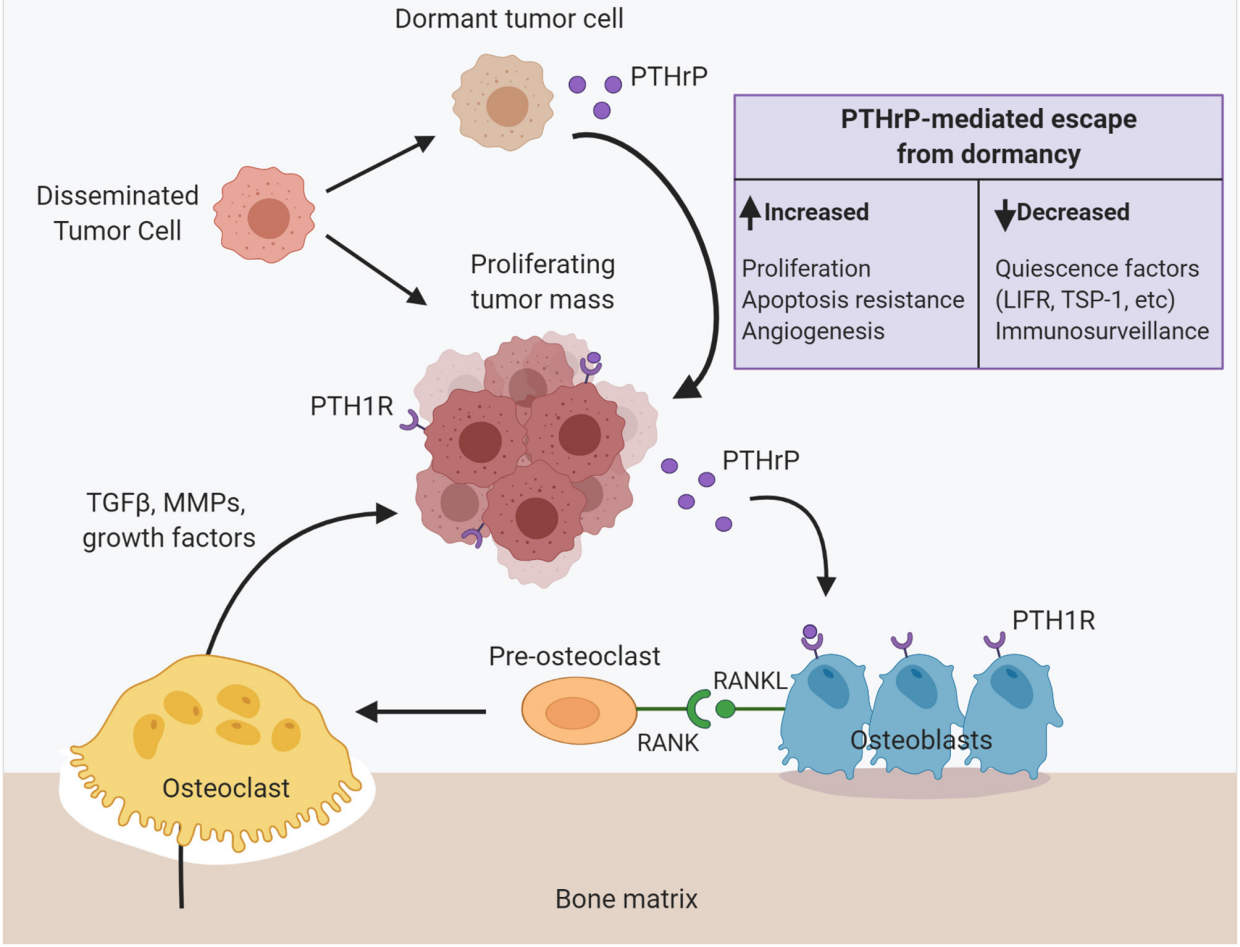
PTHrP dictates disseminated tumor cell fate in the bone to drive metastasis formation. Upon dissemination to the bone, surviving tumor cells can proliferate into a micrometastasis. Tumor cell secretion of PTHrP signals through the PTH1 receptor (PTH1R) on osteoblast lineage cells to stimulate RANKL production and osteoclastogenesis. Osteoclast-mediated resorption releases pro-tumorigenic factors from the bone matrix such as TGF-β, matrix metalloproteinases and other growth factors that further fuel tumor cell colonization, proliferation, and PTHrP production. Alternatively, disseminated tumor cells instead enter a prolonged dormant state. PTHrP drives tumor cell escape from dormancy and metastatic outgrowth via multiple mechanisms: (1) increased proliferation, (2) apoptosis resistance, (3) increased angiogenesis, (4) decreased immunosurveillance and myeloid-derived suppressor cell recruitment, (5) decreased expression of known quiescence factors (e.g., LIFR). PTHrP, parathyroid hormone-related protein; PTH1R, parathyroid hormone-related protein type 1 receptor; RANKL, receptor activator of nuclear factor–kappa B (NF_κ_B) ligand; LIFR, leukemia inhibitory factor receptor (LIFR); TSP-1, thrombospondin-1.

Given the conflicting data from both preclinical and clinical studies, a general consensus has not yet been reached regarding the role of PTHrP in tumorigenesis, metastasis, and tumor dormancy. However, there are numerous factors to consider when reconciling these findings. Stage of disease progression is critically important in this discussion. In general, the clinical data suggest that early in tumorigenesis at the primary site, PTHrP inhibits cancer growth and progression since its expression is associated with improved survival and decreased metastasis in patients with various tumor types (142, 146, 148, 153) (Figure 1). In these cases, tumor cell autonomous actions of PTHrP to alter cell proliferation may account for these findings (68). Late in disease progression, after dissemination to the bone marrow, the growth of surviving tumor cells is driven by increased PTHrP production to stimulate osteoclast-mediated bone resorption, which releases pro-tumorigenic factors that further drive tumor growth and additional PTHrP secretion (17, 18) (Figure 2). These paracrine actions of PTHrP mediated by PTH1R signaling in osteoblasts are necessary for bone metastasis growth and would explain clinical findings that PTHrP is associated with reduced disease-free survival and metastasis formation (151, 152). Lastly, the preclinical data clearly indicate that increased PTHrP expression drives breast tumor cells out of their quiescent state (28, 87, 94) *via* a mechanism independent of canonical PTH1R activation. Again, later in disease progression after long latency periods, increased PTHrP expression would favor exit from tumor dormancy in the bone and likely other metastatic sites (Figure 1). This hypothesis is supported by preclinical findings that PTHrP downregulates pro-dormancy gene expression (28), promotes proliferation, and inhibits apoptosis (61, 104) which are two key cellular responses that must be carefully balanced to regulate tumor dormancy.

As noted earlier in this review, PTHrP is a molecule with multiple biologically active domains that control its autocrine/paracrine and intracrine actions. Each of these individual actions must be considered when interpreting data on PTHrP as a dormancy regulator and prognostic factor. Preclinical studies have directly demonstrated that manipulating the expression of different PTHrP isoforms elicits markedly different biological responses. A striking example of this comes from Deftos et al. (104) where mice injected with dormant prostate cancer cells expressing the full-length PTHrP (1–173) molecule developed more extensive bone lesions than those injected with PTHrP (1–87) which lacks the full NLS, osteostatin region, and critical mitogen regulatory sequences contained in the carboxy terminus. Findings such as these can be accounted for by multiple factors. There are likely important functional elements in the region of PTHrP spanning amino acids 88–173 that uniquely promote tumor progression in bone but have not been fully elucidated. Furthermore, truncated forms of PTHrP may also assume different tertiary structures which alter binding to or interactions with other proteins that may drastically influence tumor cell behavior. Preclinical studies to further elucidate the biological activity of each PTHrP domain will be critically important to understanding the complexity of the molecule's effects in tumor development.

In interpreting findings from clinical studies on survival and prognosis in human patients, a third factor to consider is the epitope used to define positive and negative expression, as nearly all of these analyses utilize immunohistochemistry to detect PTHrP. For instance, in their work on NSCLC, Montgrain et al. (146) specifically investigated PTHrP (1–34) expression while Monego et al. (156) probed for PTHrP (109–141) and found opposite effects with regards to PTHrP as a prognostic indicator. Again, amino-terminal and carboxy-terminal PTHrP regions are known to induce disparate biological effects depending on the cell type and activation of autocrine/paracrine vs. intracrine signaling. Due to posttranslational proteolytic processing, the mature PTHrP molecule can also give rise to multiple peptides with different biological activities. Fragments encompassing the amino terminal region (residues 1–36), mid-molecule regions (38–94), (38–95), and (38–101), as well as the carboxy terminal (107–139) have been detected. Multiple peptide fragments have even been isolated from plasma (157) and urine of patients with HHM (158). Thus, antibody selection is important to consider when drawing conclusions from clinical studies relying on the immunohistochemistry to analyze expression of PTHrP and any of its cleavage products as a prognostic factor.

## PTHrP as a Therapeutic Target

Numerous studies have provided convincing evidence that PTHrP promotes tumor progression, and late recurrence by pushing tumor cells out of dormancy, resulting in poor patient survival. Thus, PTHrP would seem to be a promising therapeutic target for treating advanced human cancers. Several animal studies have demonstrated reduced distant metastasis to bone with PTHrP small molecule inhibitors (159) and neutralizing antibodies (68, 160, 161); however, human clinical data are lacking. Furthermore, there are several limitations in our current understanding of the biological activity of PTHrP that greatly complicate the development of safe and efficacious anti-PTHrP therapies at this time. PTHrP is an incredibly complex peptide with multiple distinct domains that can each influence its actions as an endocrine, paracrine, autocrine and intracrine signaling molecule. This coupled with the fact that its different isoforms and fragments can elicit diverse cellular responses could result in PTHrP targeting therapies that inadvertently promote tumor growth and recurrence if used in the wrong patient population or stage of disease progression. This is especially true in breast cancer, where preclinical and clinical data suggest that PTHrP inhibits early tumor progression, but promotes distant metastasis in advanced stages of disease (162). Studies fully defining PTHrP's role in different stages of cancer and in tumor dormancy are needed in order to identify the appropriate therapeutic window for targeting PTHrP.

In addition to direct PTHrP inhibition, alternative approaches including targeting upstream regulators of the peptide's expression have been explored. As discussed previously (20), Wnt signaling drives PTHrP expression in highly osteolytic cancer cells and thus presents a potential therapy to prevent tumor-induced bone destruction and metastatic outgrowth. However, there are challenges to targeting Wnt therapeutically due to deleterious off-target effects since signaling is critical during normal development and tissue homoestasis, especially bone formation (163–165). However, the anti-tumor activity of Wnt inhibitors has been investigated and shown varying efficacy, primarily in preclinical gastrointestinal cancer models (166, 167). There are also numerous ongoing clinical trials investigating inhibitors of the Wnt pathway in multiple other solid tumor types [NCT01351103, NCT03901950, NCT02675946, NCT03447470, NCT03395080]. In recent years, more cancer cell-specific molecular targets such as vacuolar-ATPase (v-ATPase) have been explored in the development of Wnt signaling inhibitors (168, 169). Bafilomycin and concanamycin, which directly bind to and inhibit v-ATPase, markedly inhibit Wnt/β-catenin signaling in colorectal cancer cells *in vitro* and reduce tumor cell proliferation *in vivo* without significant toxicity (168). Selective inhibitors of Porcupine (PORCN), an acyltransferase that catalyzes post-translational modification and activation of WNT ligands, have also shown promising anti-tumor activity *in vivo*, while sparing WNT-dependent tissues (170, 171). While inhibiting the Wnt pathway may be an effective therapy to decrease PTHrP expression for the treatment of metastatic cancers, more extensive investigation is needed to identify the most selective inhibitors and safest therapeutic window.

Alternative upstream targets include TGF-β which upregulates expression of Gli2 and in turn increases tumor secretion of PTHrP (172, 173). Gli2 repression significantly reduces tumor-induced bone destruction mediated by TGF-β signaling in human breast cancer MDA-MB-231 cells (172). Inhibitors against TGF-β and GLI proteins have been evaluated in clinical trials as anti-cancer therapy (174) [clinicaltrials.gov]. Another study demonstrated that the EGF receptor promotes PTHrP production, since treatment with erlotinib, an EGF receptor tyrosine kinase inhibitor, suppresses PTHrP expression in non-small cell lung cancer cells and reduces osteolysis (175). Other EGF receptor tyrosine kinase inhibitors including gefitinib also reduce PTHrP levels (176). Lastly, targeting downstream effectors of PTHrP may also provide an efficacious strategy. For instance, as mentioned previously, PTHrP (1–139) overexpression in MCF7 cells also represses expression and downstream signaling of LIFR, a known breast tumor suppressor and dormancy factor in the bone (28). Consequently, LIFR downregulation promotes human MCF7 breast cancer cell emergence from dormancy in the bone. Treatment with the histone deacetylase inhibitor valproic acid subsequently increases LIFR expression in human MCF7 breast cancer cells *in vitro*, suggesting that targeting LIFR, a downstream factor in PTHrP signaling, may effectively maintain tumor cells in a dormant state to prevent metastatic outgrowth. Multiple strategies should therefore be considered in order to develop the most selective and effective PTHrP targeting therapies.

## Conclusions

PTHrP is a unique multifunctional protein with diverse effects on tumor cell behavior mediated by its different biological domains and isoforms that arise from posttranslational processing. Overall, preclinical and clinical studies suggest that PTHrP inhibits tumor progression in early stages of disease while it functions in the opposite manner to promote tumor development and metastasis formation in advanced cancers, resulting in diminished survival in patients. This is especially true in the bone, a common site of metastasis, where PTHrP-mediated osteolysis is critical for tumor cells to establish as colonies and grow. Furthermore, while the studies are still limited, an important role for PTHrP in promoting tumor emergence from a dormant state is an emerging area of interest. Despite its complexity, more studies that fully uncover the unique biological activities of PTHrP and its domains that regulate its endocrine, autocrine, paracrine, and intracrine signaling could uncover numerous additional targets to explore as anticancer therapeutics.

## Author Contributions

CE drafted the manuscript. CE and RJ edited the manuscript. All authors contributed to the article and approved the submitted version.

## Conflict of Interest

The authors declare that the research was conducted in the absence of any commercial or financial relationships that could be construed as a potential conflict of interest.
